# The Second National Ballistics Imaging Comparison (NBIC-2)

**DOI:** 10.6028/jres.119.028

**Published:** 2015-01-08

**Authors:** TV Vorburger, J Yen, JF Song, RM Thompson, TB Renegar, A Zheng, M Tong, M Ols

**Affiliations:** National Institute of Standards and Technology, Gaithersburg, MD 20899; Bureau of Alcohol, Tobacco, Firearms and Explosives (ATF), Ammendale, MD 20705

**Keywords:** ballistics identification, forensic science, NBIC, NIBIN, standard bullet, standard cartridge case, standard reference material

## Abstract

In response to the guidelines issued by the American Society of Crime Laboratory Directors/Laboratory Accreditation Board (ASCLD/LAB-International) to establish traceability and quality assurance in U.S. crime laboratories, NIST and the ATF initiated a joint project, entitled the National Ballistics Imaging Comparison (NBIC). The NBIC project aims to establish a national traceability and quality system for ballistics identifications in crime laboratories utilizing ATF’s National Integrated Ballistics Information Network (NIBIN). The original NBIC was completed in 2010. In the second NBIC, NIST Standard Reference Material (SRM) 2461 Cartridge Cases were used as reference standards, and 14 experts from 11 U.S. crime laboratories each performed 17 image acquisitions and correlations of the SRM cartridge cases over the course of about half a year. Resulting correlation scores were collected by NIST for statistical analyses, from which control charts and control limits were developed for the proposed quality system and for promoting future assessments and accreditations for firearm evidence in U.S. forensic laboratories in accordance with the ISO 17025 Standard.

## 1. Introduction

In the late 1990s, the National Integrated Ballistics Information Network (NIBIN), initially called the Ceasefire Program, was established in the United States [1,2]. The NIBIN consists of about 150 data acquisition stations for acquiring and reviewing fired cartridge cases and bullets submitted to federal, state, and local crime labs during the course of criminal investigations. The NIBIN is supported and coordinated by the Bureau of Alcohol, Tobacco, Firearms, and Explosives (ATF) and uses a technology known as the Integrated Ballistics Identification System (IBIS^1^) [3] manufactured by Forensic Technology Inc., now part of Ultra Electronics. Each station consists of an automated optical microscope with special fixtures for cartridge cases and bullets and an interface to a regional database of ballistics images, with the capability to correlate automatically one’s own images with entries in the regional database using correlation software. To establish measurement traceability and quality assurance in expectation of laboratory assessments using the ISO 17025 standard [4], the National Institute of Standards and Technology (NIST) in collaboration with the ATF developed Standard Reference Material (SRM) 2460 Bullets [5–7] and 2461 Cartridge Cases [8]. NIST also developed profiling (2D) and areal (3D) measurement systems for surface topography [7,8] as well as correlation software for quantifying the similarity of topography profiles and images using NIST proposed correlation parameters [5].

In recognition of the guidelines [9] issued by the American Society of Crime Laboratory Directors/Laboratory Accreditation Board (ASCLD/LAB-International) to establish traceability and quality assurance in U.S. crime laboratories, NIST and ATF in cooperation with a number of crime laboratories have recently completed two joint projects known as the National Ballistics Imaging Comparison (NBIC) and NBIC-2. The aim of these projects is to establish a national traceability and quality system using the SRM bullets and cartridge cases as check standards for NIBIN acquisitions and correlations. The physical standards and established procedures can be used for detecting and exploring any quality problems that may arise from operators’ acquisition procedures or from IBIS software and networks. Thirty one experts and 18 U.S. crime laboratories participated in one or both of these projects. They each took a number of acquisitions of NIST SRM 2460 Standard Bullets and 2461 Standard Cartridge Cases over the course of about a year, and the acquired images were correlated with Golden Images at the National Laboratory Center of ATF, from which control charts and control limits have been developed. These procedures will enable the development of a traceability and quality system for NIBIN and U.S. crime laboratories in expectation of assessments by ASCLD/LAB [9] and other organizations in accordance with the ISO 17025 Standard [4].

The original NBIC was completed in 2010 and is discussed in a previous paper [10]. The NBIC project was carried out entirely on IBIS Heritage Model workstations. Since the completion of NBIC, Forensic Technology, Inc. developed a new technology known as BRASSTRAX for acquisition of cartridge cases. IBIS BRASSTRAX technology is designed to be interoperable with IBIS Heritage technology. All the same, we initiated a new project known as NBIC-2 to test whether the procedures and conclusions reached during the original NBIC project needed to be modified for stations running BRASSTRAX technology. In this report we discuss the results for NBIC-2. Sections 2 to 5 include discussions of the NIST SRM Standard Bullets and Cartridge Cases, the NIST 2D and 3D topography measurement system, and an overview of the NBIC Project. In Secs. 6 to 8 we discuss results obtained from NBIC-2. In Sec. 9, we discuss a proposed system for quality assurance of NIBIN acquisitions and correlations and include an initial uncertainty estimate for certain NIBIN acquisitions.

## 2. Standard Reference Materials 2460 Standard Bullets and 2461 Standard Cartridge Cases

The SRM 2460 Bullets and 2461 Cartridge Cases were developed as reference standards for crime laboratories 1) to help verify that the computerized optical imaging equipment for bullets and cartridge cases is operating properly and 2) to establish quality assurance and traceability in accordance with ISO 17025 [4].

**Fig. 1:**
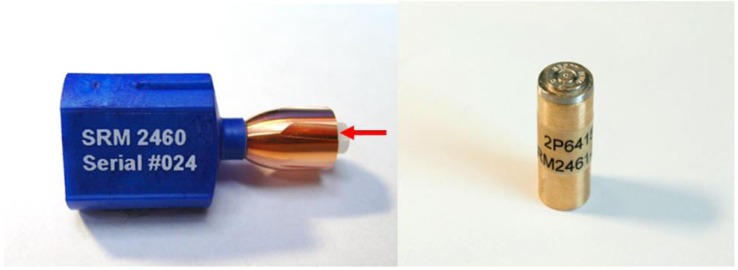
A SRM 2460 Standard Bullet (left) and a SRM 2461 Standard Cartridge Case (right). The red arrow indicates one of six land engraved areas around the periphery of the standard bullet.

**Fig. 2:**
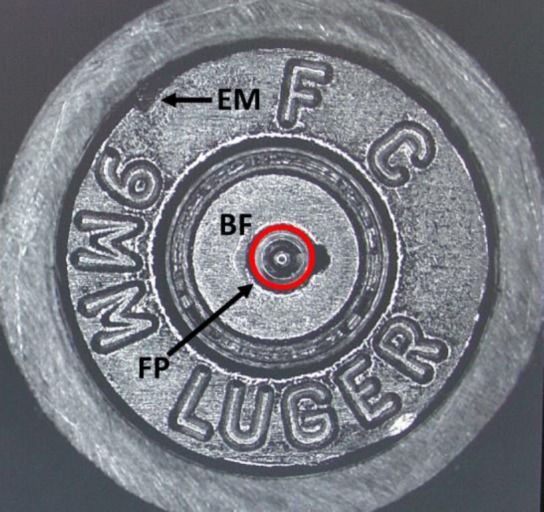
Photograph of the base of a SRM 2461 standard cartridge case indicating the breech face impression (BF), firing pin impression (FP), and ejector mark (EM).

Units of SRM 2460 and 2461 are shown in Fig. 1. Thirty five units of SRM 2460 and 137 units of SRM 2461 (serial numbers ranging from 106 to 278 with some gaps in the numbering) were produced, measured, certified, and made available. Each of these units is nominally identical to the other units, and optical images of the key areas of the surfaces of one unit should be nearly identical to comparable images from all the other units, as well as to master images stored and maintained in the database by the ATF. These master images are called “Golden Images.” The key areas of the bullet are the six land engraved areas (LEAs) spaced around the bullet periphery. One of these LEAs is clearly shown in Fig. 1. The key areas of the cartridge case are shown in Fig. 2. In order to protect the outer surface of the SRM cartridge case, the diameter of the brass cylinder (about 12.7 mm) is made larger than the diameter of the cartridge case (about 9 mm). All these areas have been measured for topography at NIST, and their IBIS images have been acquired at the ATF’s National Laboratory Center in Ammendale, MD. The fabrication methods for the SRM Bullets and Cartridge Cases and the reproducibility of the individual units have been described elsewhere [5,11].

**Fig. 3:**
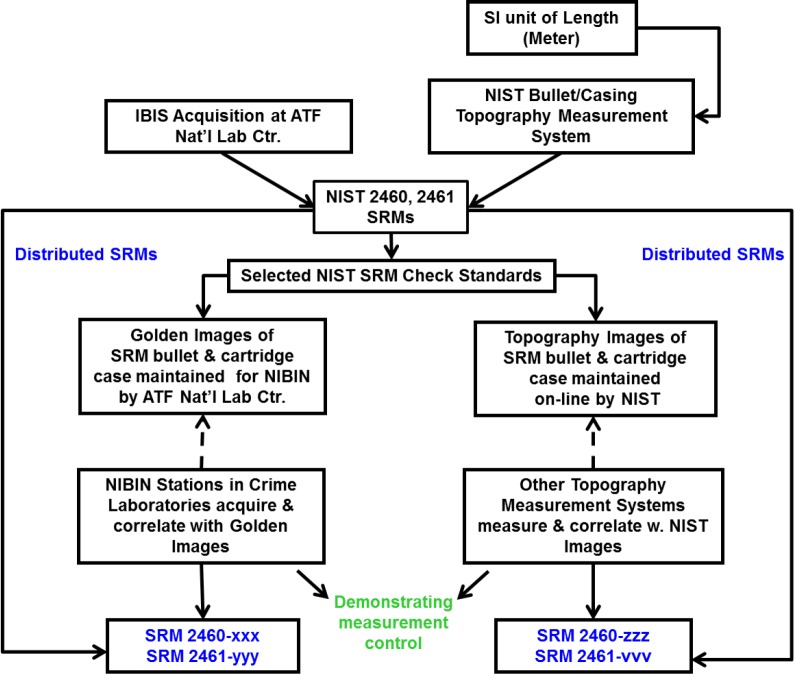
Schematic diagram of two types of quality control chains maintained by NIST and ATF for SRMs 2460 and 2461.

There are two ways to use these SRMs for quality control, as shown schematically in Fig. 3. Topography profiles of the six LEAs on the SRM bullet masters and topography images of the three regions on the casing masters are available on the Internet [12,13] and may be correlated by users to topography profiles and images acquired on their own topography measurement systems. This enables users in crime labs to demonstrate the accuracy of their systems for measuring topography of bullets or cartridge cases and then estimating similarity between pairs of exhibits. Correlation coefficients determined by NIST for these topography profiles and images are quoted on the certificates that accompany SRMs 2460 and 2461 [14,15].

Alternatively and more commonly, images of a standard bullet or cartridge case obtained by crime labs using the IBIS system may be compared to the Golden Images using IBIS correlation software. Control values for both bullets and cartridge cases were developed under the first NBIC project, which involved testing of the SRMs over about one year by 19 IBIS operators working on 13 different IBIS stations. In the meantime, a new version of IBIS hardware and software has been developed by the manufacturer, specifically for acquisition and analysis of cartridge cases, and is being disseminated to the NIBIN system and elsewhere. The new version, called BRASSTRAX [16], uses topographic contrast to display 3D images of cartridge case surface regions. In addition, optical images are obtained in a manner similar to the previous IBIS Heritage version except at twice the pixel resolution. Mainly for this reason, it is important to check whether the control values obtained by NBIC for the cartridge cases have changed due to the change in pixel resolution or to incidental changes with respect to the IBIS Heritage version. Hence, the NBIC-2 was initiated to determine new control values for correlations obtained with BRASSTRAX on the three cartridge case regions or, alternatively, to affirm the original control values obtained by NBIC. The correlation values thus obtained are traceable to one of the Golden Images maintained by the ATF. Bullets were not used for NBIC-2 because the BRASSTRAX is designed only for acquisition of cartridge cases.

## 3. NIST and ATF Measurements and the Golden Images

**Fig. 4:**
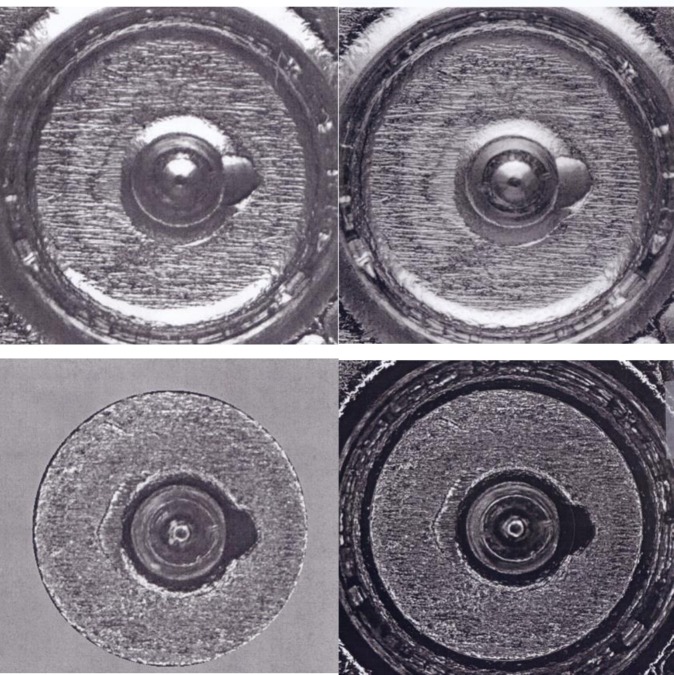
Left – Golden Images of the breech face impression for SRM 2461 S/N 006 obtained with an IBIS Heritage station at ATF’s National Laboratory Center, May 16, 2008. Right – Golden Images of the breech face impressions of SRM 2461 S/N 186 obtained with the BRASSTRAX on October 26, 2010. Upper – taken with oblique lighting from the 6 o’clock direction; lower – taken with ring lighting.

**Fig. 5:**
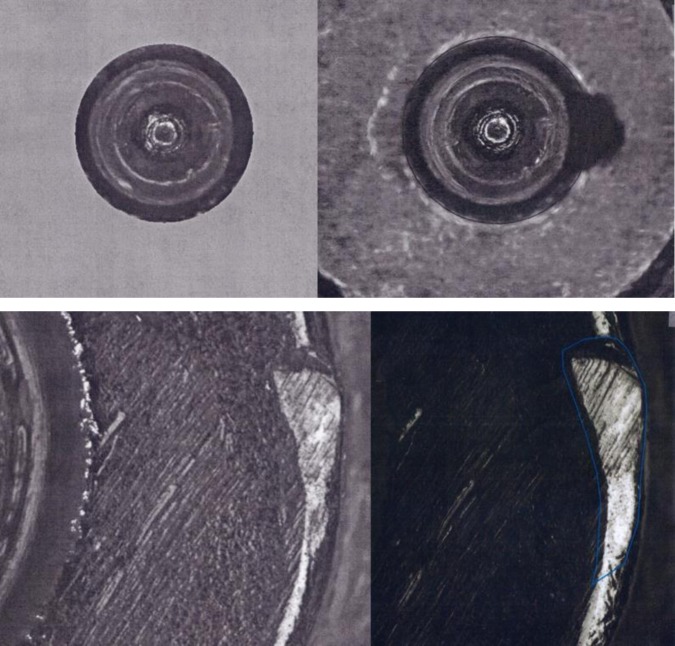
Left – Golden Images of the firing pin impression of SRM 2461 S/N 017 (top left) and ejector mark of SRM 2461 S/N 015 (bottom left) obtained with an IBIS Heritage station at ATF’s National Laboratory Center, May 16, 2008; Golden Images of the firing pin impression of S/N 111 (top right) and the ejector mark of S/N 143 (bottom right) with the BRASSTRAX-3D (right) on October 26, 2010.

The NIST system for measuring and correlating surface profiles and topography images has been described previously [7,8,10]. The optical images that function as the Golden Images for the BRASSTRAX stations in the NIBIN were acquired by NIST and ATF personnel using the BRASSTRAX station at the ATF National Laboratory Center. They were selected from a large set of SRM images as the best examples of IBIS images for breech face impressions, firing pin impressions, and ejector marks by virtue of their high correlation scores with respect to the images of other units of SRM 2461. The Golden Image for the breech face impression was selected from unit 186 of SRM 2461, that for the firing pin impression from unit 111, and that for the ejector mark from unit 143. These BRASSTRAX Golden Images are shown on the right hand sides of Figs. 4 and 5. By comparison, the Golden Images applicable to the IBIS Heritage stations are shown on the left hand sides of Figs. 4 and 5. These were obtained in 2008 from prototype units 006, 017, and 015 of SRM 2461.

A schematic diagram for the quality system using the SRM bullets and cartridge cases is shown in Fig. 3. All of the SRMs were measured at NIST for their topography, and their reflectance images were acquired on the IBIS Heritage station at the ATF National Laboratory Center. In addition, all of the SRM cartridge cases were re-acquired with the ATF’s BRASSTRAX station there. NIST maintains the master topography images of the bullets and cartridge cases on its Websites, [12,13]. The ATF maintains the reflectance images on one of the NIBIN servers. Users of IBIS stations with access to the NIBIN network can compare their acquired images of SRM 2460 and SRM 2461 with the ATF’s Golden Images, and users of topography imaging systems can compare their topography images of SRM 2460 and SRM 2461 with the corresponding images on the NIST website.

**Fig. 6:**
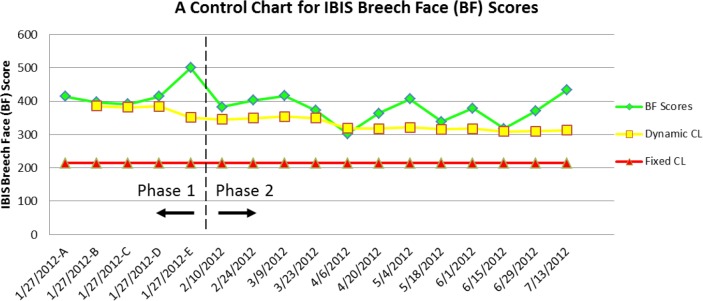
Example of a control chart generated during NBIC-2. Both a fixed control limit and a dynamic control limit are plotted along with a time series of breech face correlation scores.

Figure 6 shows a control chart for correlations of the images of the breech face impression of one unit of SRM 2461 acquired during NBIC-2. The top line shows the correlation scores for IBIS BRASSTRAX images with respect to the Golden Image obtained with the ATF’s BRASSTRAX station in Ammendale MD. The scores are unitless numbers resulting from the proprietary correlation software of the BRASSTRAX manufacturer, Forensic Technology, Inc. The higher the score, the stronger the correlation. The bottom line represents the specified control limit of 217 for breech face (BF) scores, which was calculated from the results of the NBIC-1 project. The middle line is a “dynamic control limit” (Dynamic CL) based on the measured data, which has been proposed and used by NIST for measurement assurance of surface calibrations [17]. The dynamic control chart will be further discussed in Sec. 6. Phase 1 on the chart indicates a period of repeatability tests all acquired on the same day. Phase 2 represents a longer period of testing over approximately 5 months.

As stated earlier, SRM cartridge case units 186, 111, and 143 were used to create the master Golden Images at the ATF. After the topography measurements at NIST, all the SRM cartridge cases were acquired at the National Laboratory Center of ATF using their BRASSTRAX station under standard operating conditions [18]. A set of the best images with the highest correlation scores with respect to the other units was selected as the set of Golden Images (see Figs. 4 and 5). By acquiring images of the SRM cartridge cases at local and state IBIS sites, and correlating the images with the Golden Images, differences in IBIS operating conditions between the local and state IBIS sites and the ATF National Laboratory Center can be detected. This method, therefore, enables the images at local IBIS sites to be traceable to the Golden Images of ATF’s National Laboratory Center. A similar approach was previously developed for SRM bullets [10].

## 4. The National Ballistics Imaging Comparison (NBIC) Projects

The project goal for both NBIC projects is to establish a quality system for ballistics signature correlations in U.S. crime laboratories within the NIBIN. The original NBIC project was focused on the IBIS Heritage model system, the most widely used at NIBIN sites. Both SRM 2460 bullets and 2461 cartridge cases were used as the reference standards. By repeating tests of the SRM bullets and cartridge cases at local and state IBIS sites, and correlating the images with the Golden Images established at the ATF, control charts and control limits were developed for quality assurance of ballistics acquisitions and correlations in NIBIN. Use of these procedures enable the development of a traceability and quality system for NIBIN and U.S. crime laboratories for use in assessments by ASCLD/LAB [9] and other organizations in accordance with the ISO 17025 Standard [4]. This system can also be used for detecting and exploring any quality problems arising from operators’ acquisition procedures, IBIS stations and correlation servers, as well as from the SRM standards themselves.

The protocol called for 24 acquisitions of both a SRM bullet and a cartridge case for each participant over the course of about a year. The correlation scores were entered on a spreadsheet designed by NIST for statistical analyses, from which control charts and control limits were developed for the proposed traceability and quality system. Nineteen ballistics examiners from 13 IBIS sites participated in this project from July 2008 to March 2010. After statistical analysis, draft control charts and control limits were developed for the proposed quality system. A report on the NBIC Project was published in 2012 [10].

## 5. The Second National Ballistics Imaging Comparison Project (NBIC-2)

The original NBIC project was performed using IBIS Heritage stations. Subsequently a new technology was introduced known as BRASSTRAX. The new technology was targeted for improved quality and manipulation of cartridge case images and, among other features, included a system upgrade, a camera with double the pixel resolution of the IBIS Heritage, and the capability to create topography images of cartridge case surfaces that could be viewed from different directions. The principal mode of correlation, like IBIS Heritage, was still based on the 2D reflectance images. We, therefore, initiated the NBIC-2 project to determine whether the control limits developed for NBIC should be adjusted, mainly due to the system upgrade and the improved camera resolution.

All participants agreed to use their BRASSTRAX stations for image acquisitions of their SRM cartridge case and for correlations of those images with respect to the BRASSTRAX Golden Images. The protocol called for 17 acquisitions of the SRM cartridge case for each participant over the course of about six months. Fourteen ballistics examiners from 11 IBIS sites participated in this project from November 2011 to August 2013. The first set of test data was sent to NIST in 2012. Based on the data analyses, a feedback report was presented at a NIST/ATF workshop held in Phoenix AZ on August 27–28, 2013.

The correlation scores were entered on a spreadsheet designed by NIST for statistical analysis. When the correlation scores are entered in the spreadsheet, a control chart with dynamic and fixed control limits is automatically generated. A typical control chart of results for one of the participants is shown in Fig. 6. It contains correlation scores for 17 image acquisitions of the breech face region in chronological order. The fixed control limit was obtained from the results for NBIC-1. The dynamic control limit [17] is a running value derived from the mean (*µ*) and standard deviation (*σ*) of the accumulating scores; it corresponds approximately to a one-sided 95 % confidence level. When a correlation score falls below the dynamic control limit, it serves as an early warning, independent of the fixed control limit, that the acquisition station might be undergoing significant drift or losing accuracy in other ways.

After data was received from all participants, statistical analysis was performed and overall control limits were developed for the data in each region of the SRM cartridge case. These are described next.

## 6. Statistical Analysis and Control Limits for the NBIC-2 Project

**Fig. 7:**
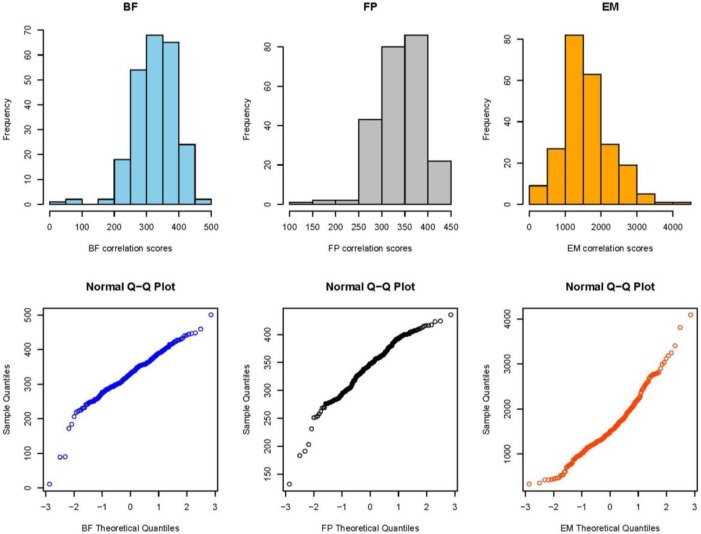
(Top) the collective distribution of BRASSTRAX correlation scores vs. their NIST/ATF Golden Images for 14 examiners for breech face (BF), firing pin (FP), and ejector mark (EM). (Bottom) Q-Q plots [19,20] for the same data. The correlations were performed with respect to the BRASSTRAX Golden Images (see right sides of Figs. 4 and 5) housed in the Region 6 Server of the NIBIN at the ATF National Laboratory Center, Ammendale, MD.

Figure 7 (top) shows the collective distribution of scores for correlations of breech face (BF) impressions, firing pin (FP) impressions, and ejector marks (EM) of the SRM cartridge cases for 14 operators using 11 different BRASSTRAX stations. Since each operator recorded 17 readings for each of the three regions, there are about 238 readings in each histogram. The distributions for these finite samples each seem to be close to a Gaussian distribution. An alternative procedure for showing fit to a Gaussian distribution is shown by Fig. 7 (bottom). Here the data are plotted on a Normal Q-Q plot [19,20] with the lowest data value at the lower left and the highest data value at the upper right. If the histogram is Gaussian, then the Q-Q plot of the data is a straight line. The graphs seem to show this straight-line behavior except for points at the extreme ends of the data.

The above analysis gives us confidence that, despite possible differences between operators, the overall pooled data for each region form essentially a single population of scores and that a Gaussian statistical model is a good approximation for further analysis. We now estimate consensus control limits for each of the distributions to serve as guidelines to future users of the SRM cartridge cases as control standards. When correlation scores for BRASSTRAX images fall below a control limit, users should investigate whether their acquisition station is operating correctly or is changing in some way. Possible problem scenarios include:

- Instrument changing and going out of calibration,
- Operator not following proper procedure,
- Physical standard itself becoming contaminated or flawed.

In Sec. 7, we will also use the control limits to investigate the above data to determine if there are station-related problems causing any of the lower scores in the distributions. In the previous NBIC-1 comparison, which was performed with both SRM bullets and prototype SRM cartridge cases, scores falling below the control limits led us to findings of the following quality problems [10]:

- In some cases, the proper alignment procedure for acquiring bullets was not being followed.
- For two prototype cartridge cases, contamination of the firing pin region was taking place. This led us to change the manufacturing procedure to produce the final SRM cartridge cases used in the current NBIC-2 comparison.
- A bug in the software for transfer of ejector mark images between regions in the NIBIN was leading to incorrect correlation scores for ejector marks. The root cause of this problem was then diagnosed by the vendor who performed a modification in the software to eliminate the bug.

To establish the control limits we only used the data taken during Phase 2, the relatively long term part of the investigation, not the shorter term Phase 1 data. The length of time over which the Phase 2 data were taken is comparable to the length of time envisioned for a potential user’s control chart, whereas the Phase 1 data were taken over a much shorter time period. Therefore, we expect that the Phase 2 data would show statistical variability more comparable to that of a control chart than the Phase 1 data. Furthermore, the Phase 1 data could show initial short term trends not present in the Phase 2 data as the operators became accustomed to the acquisition procedure for the SRM cartridge cases. Both characteristics make Phase 1 data less relevant than the Phase 2 data for determining control limits in future control charts.

We also did not use data from Phase 2 that fell below the apparent straight lines on the Q-Q plots. These points were considered to be outliers from their respective populations and were investigated further for possible quality issues. The points that were discarded this way include one point from the breech face region and three points from the firing pin region. The images associated with these points will be discussed later in Sec. 7.

**Fig. 8:**
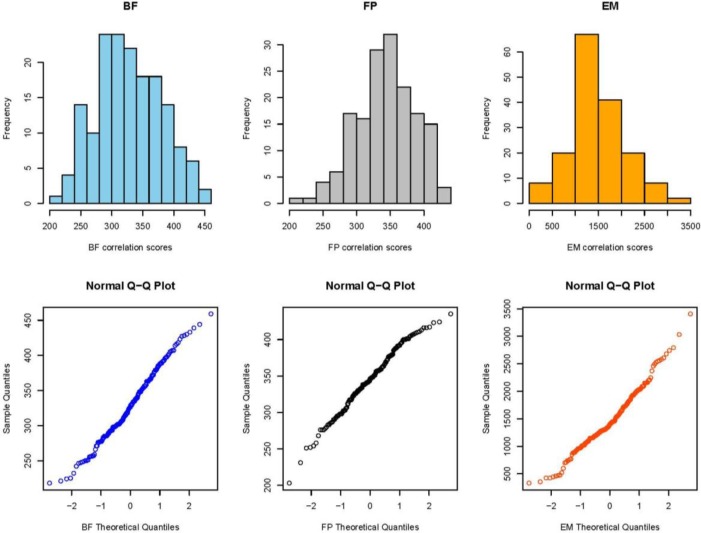
(Top) the collective distribution of BRASSTRAX correlation scores for images of SRM 2461 units obtained by 14 examiners for breech face (BF), firing pin (FP) and ejector mark (EM). (Bottom) Q-Q plots for the same data. The correlations were performed with respect to the BRASSTRAX Golden Images (right sides of Figs. 4 and 5) housed in the Region 6 Server of the NIBIN at the ATF National Laboratory Center, Ammendale, MD. The data plotted exclude data in Fig. 7 from Phase 1 and data judged to be outliers.

The remaining data used to establish the control limits are plotted in Fig. 8. Assuming that the distributions of scores are Gaussian for each of the regions, a one sided control limit (CL), which controls the low values only, with a 95 % confidence level was calculated as

CL=(μ−1.645σ), (1)

where *µ* and *σ* represent the collective mean and standard deviation, respectively. The factor 1.645 is the *t*-factor for a Gaussian distribution corresponding to a one sided 95 % confidence level for the control of low IBIS scores.

Table 1 shows the control limits for correlation scores obtained from the Phase 2 images of all three cartridge case regions obtained with BRASSTRAX. The right hand column shows the control limits obtained earlier during NBIC-1 using the IBIS Heritage acquisition stations. Table 1 shows slightly higher control limits in NBIC-2 for the breech face and ejector mark regions; the difference between the two control limits amounts to less than one standard deviation. However, the control limit from NBIC-2 for the firing pin region is higher than that for NBIC-1 by more than two standard deviations.

**Fig. 9:**
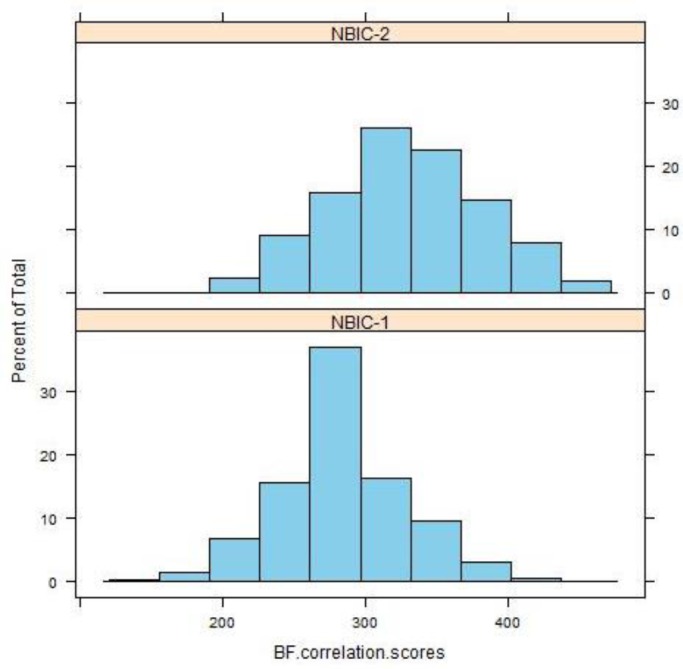
Histograms of correlation scores for SRM 2461 breech face impressions for NBIC-1 and NBIC-2. The unit along the vertical axis is now the percentage of the total number of observations, rather than “Frequency”, the unit used in Figs. 7 and 8.

The differences in the control limit are directly attributable to overall increases in the correlation scores of NBIC-2 relative to NBIC-1. Figures 9^11^ show histograms of scores for all regions for both NBIC-1 and NBIC-2. Overall increases are apparent for all three regions of the cartridge cases, a minor one for the breech face impressions and a major one for the firing pin impressions. The ejector marks show a minor increase in the control limit but a large increase in the spread of the distribution. The correlation software for ejector marks apparently can produce very high scores for particularly favorable matches.

The scores and control limits for firing pin impressions can differ significantly even though no modification to the correlation software had been declared by the manufacturer between NBIC-1 and NBIC-2. One possibility for the difference is that the pixel resolution of NBIC-2 images is twice as high as that for NBIC-1. Hence the images could provide more detail than before. Indeed, Fig. 5 (top) shows that the Golden Image for firing pin obtained during NBIC-2 appears to have more contrast and slightly better sharpness than the comparable Golden Image obtained during NBIC-1.

## 7. Quality Issues Uncovered by the Control Charts for NIBIN Acquisitions and Correlations

Three major sources of uncertainty to be considered for NIBIN acquisitions and correlations are:

1. Operator, acquisition process, and IBIS acquisition hardware including the optical microscope. These factors are grouped together because it is more straightforward to calculate their combined effects from the data than it is to separate the individual components.
2. IBIS correlation software and the NIBIN correlation network.
3. Calibration and reference standards including the SRM bullets and cartridge cases.

The NBIC-1 project uncovered procedural issues for certain individual bullet acquisitions, striking anomalies in the correlation scores for ejector marks, and contamination on the surfaces of at least two of the prototype cartridge cases. The anomalies for ejector marks led to discovery of a bug in the transfer protocol between regions for ejector mark images, which was subsequently repaired. The contamination was addressed by changing the manufacturing process for the final SRM 2461 product.

In the NBIC-2 project, we selected some questionable IBIS correlations from the control charts and analyzed their corresponding images. The questionable results all seemed to involve imaging, alignment, and illumination issues. In ballistics image acquisition, lighting conditions include the type of light source, the light direction, and the intensity, and other imaging issues include the color and reflectivity of the material and the image contrast. The lighting conditions can be easily controlled when performing pairwise comparisons in a comparison microscope, but they are not so easy to maintain and reproduce when the images are acquired individually on an automated acquisition station. These issues have a significant effect on imaging quality and correlation scores, and should be standardized and well controlled. Even for the standardized, automated lighting conditions used in the IBIS microscopes [18], variations caused by the measurement setup and acquisition process may significantly affect signature acquisitions and correlations. Although the questionable results are ascribed to lighting conditions, in all but one case our diagnoses have stopped short of locating a root cause.

**Fig. 12:**
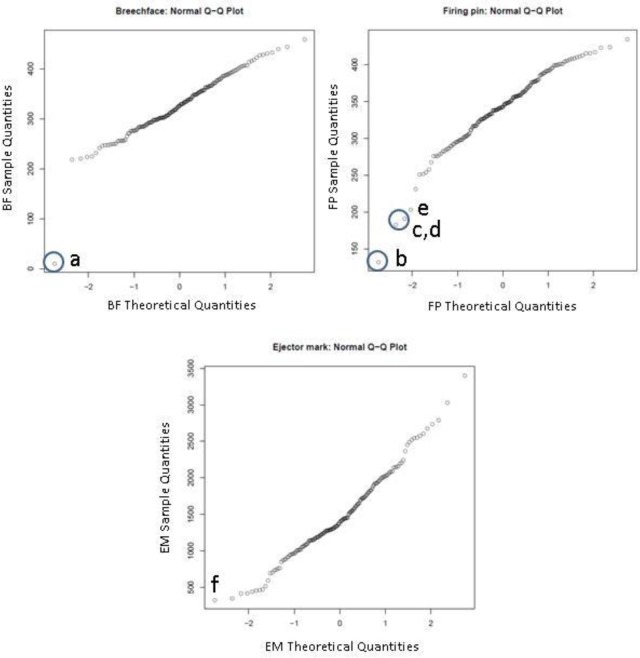
Q-Q plots for Phase 2 data of NBIC-2. The circled points (a,b,c,d) were not included in the populations used to calculate the control limits and are not shown in Fig. 8. Images for the points e, f are also discussed here.

**Fig. 13:**
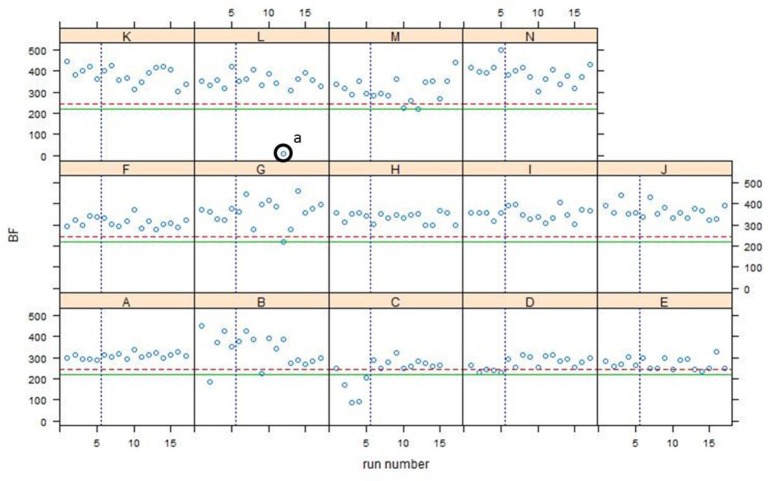
Control charts of all correlation data for breech face impressions acquired by 14 NBIC-2 participants, indicated by capital letters. The dashed straight line is the proposed control limit for BRASSTRAX acquisitions for the SRM 2461 breech face region. The solid straight line is the control limit obtained by NBIC-1 using IBIS Heritage technology. The vertical dotted line separates the Phase 1 and Phase 2 data.

**Fig. 14:**
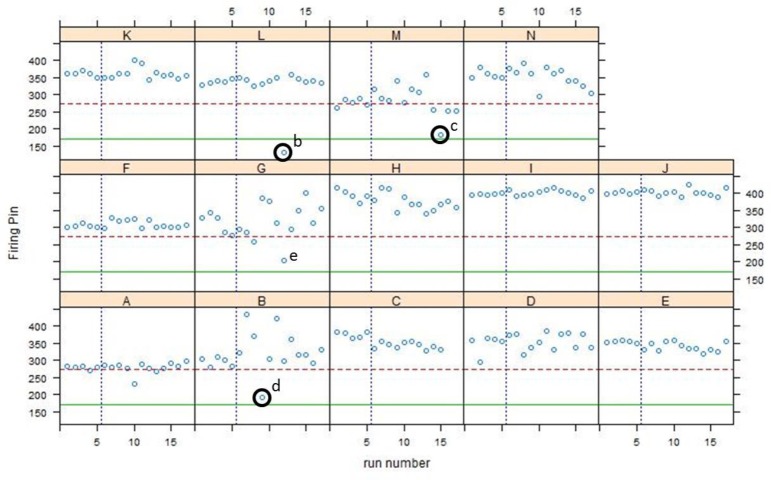
Control charts of all correlation data for the firing pin region acquired by 14 participants in NBIC-2. The dashed straight line is the proposed control limit for BRASSTRAX acquisitions for the SRM 2461 firing pin region. The solid straight line is the control limit obtained by NBIC-1 using IBIS Heritage technology.

**Fig. 15:**
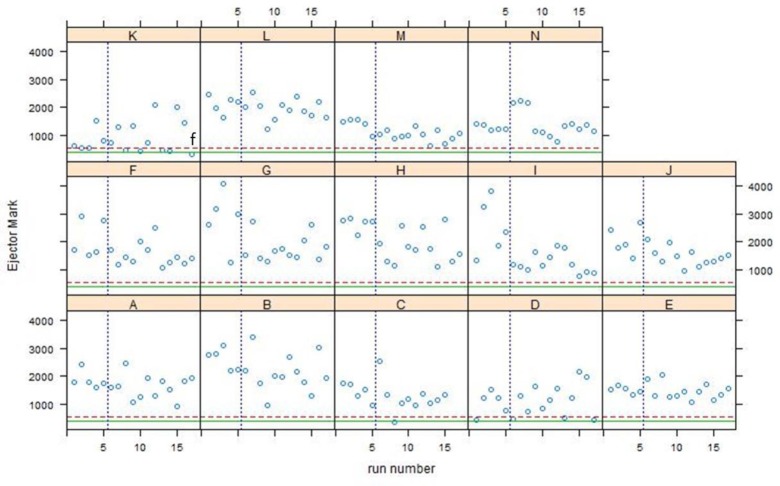
Control charts of all correlation data for the ejector mark region acquired by 14 participants in NBIC-2. The dashed straight line is the proposed control limit for BRASSTRAX acquisitions for the SRM 2461 ejector mark region. The solid straight line is the control limit obtained by NBIC-1 using IBIS Heritage technology.

**Fig. 16:**
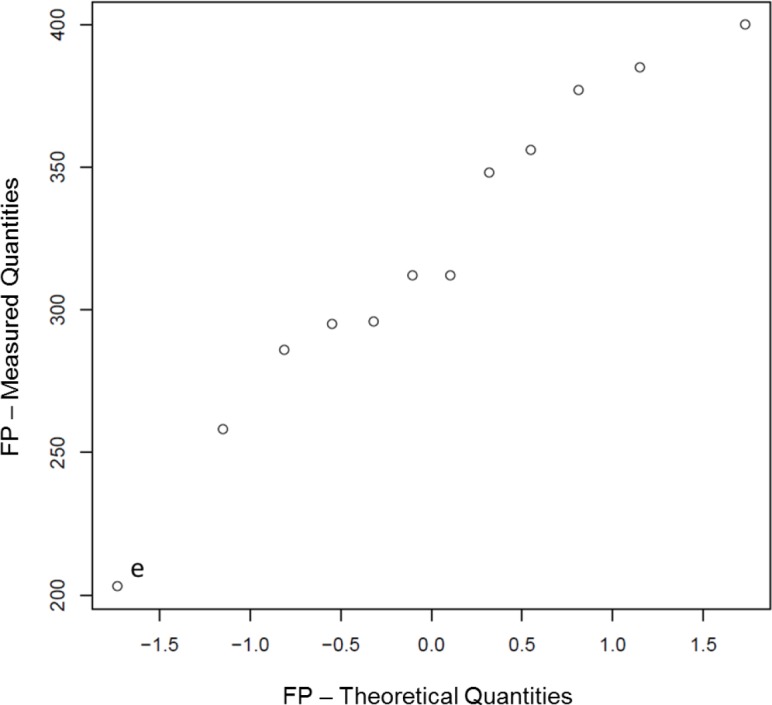
Q-Q plot for the Phase 2 data for Station G in the NBIC-2 study.

Q-Q plots for all Phase 2 data are shown in Fig. 12 and control charts for all 14 participants are shown in Figs. 13^15^. The NBIC-1 and NBIC-2 control limits are also shown. The data points in Phase 2 that were not plotted in Fig. 8, because they seemed to fall below straight lines in the Q-Q plots, are highlighted by circles and labelled a–d. These points were investigated by inspecting the acquired images to determine if there were flaws in the images arising from problems with the acquisitions. In addition, points in Figs. 12, 14, and 15 labelled e and f were also investigated. Although point e looks fairly low on the Q-Q plot of Fig. 12, we included it in the control-limit analysis because that data point seems fairly consistent with the other data points in the specific Q-Q plot for that station and operator, shown in Fig. 16.

### 7.1 Breech Face Impression

**Fig. 17:**
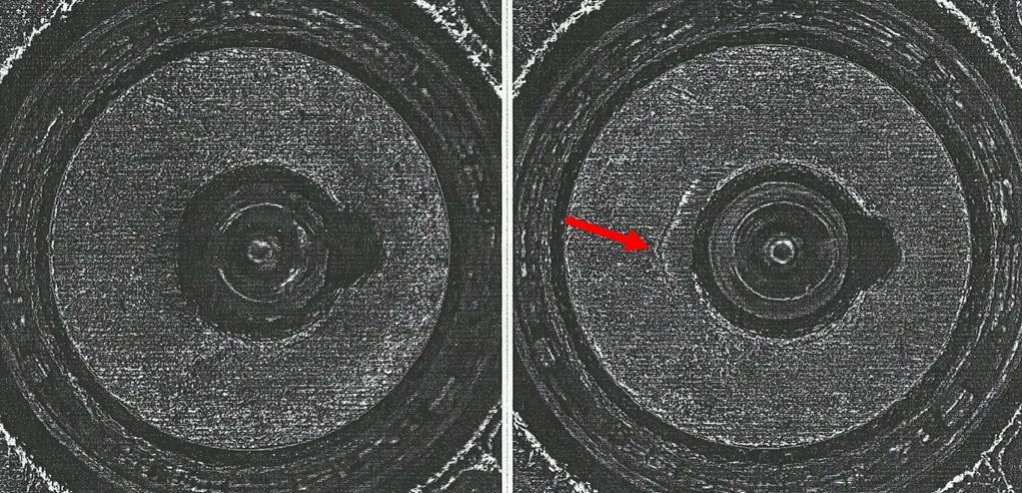
NIBIN image corresponding to point a in Fig. 12 and 13 (left) and Golden Image for breech face (right).

As shown in Figs. 12 and 13, there was one low correlation score for breech face in Phase 2, designated as “a”. The breech face image used for the correlation is shown on the left in Fig. 17 next to the corresponding Golden Image on the right. The two images are fairly similar; however, faint shadows are apparent in the left hand image, and the area of apparent pushed up material designated by the arrow in the Golden Image is washed out in the left hand image. We do not know what caused the changes in the left hand image. One can hypothesize an illumination, alignment, or focus problem, but one was not recorded at the time. This faulty imaging condition was only temporary because previous and subsequent correlation scores for this station and this operator were in the normal range. However, the low correlation score here for the SRM breech face acquisition with respect to the Golden Image does reveal an acquisition problem that is substantiated by inspection of the images themselves.

### 7.2 Firing Pin Impressions

**Fig. 18:**
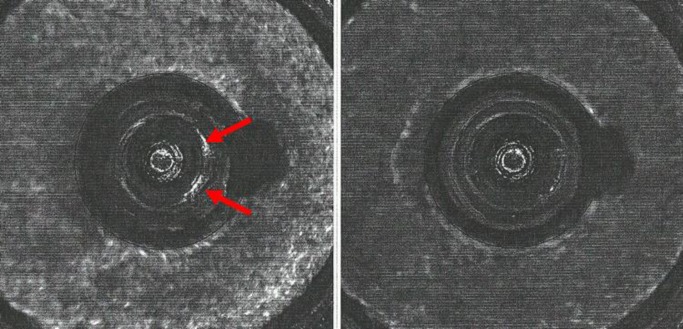
NIBIN image (left) corresponding to point b in Fig. 12 and 14 and Golden Image for firing pin (right).

**Fig. 19:**
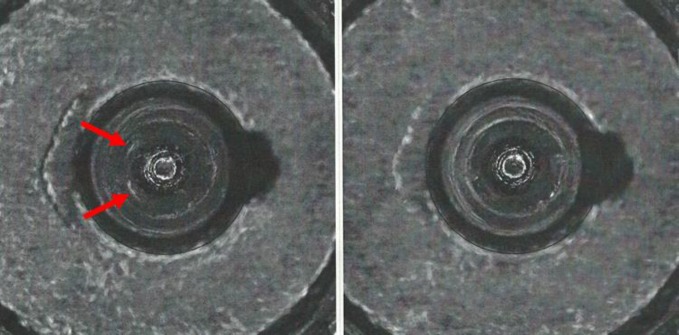
NIBIN image (left) corresponding to point c in Fig. 12 and 14 and Golden Image for firing pin (right).

**Fig. 20:**
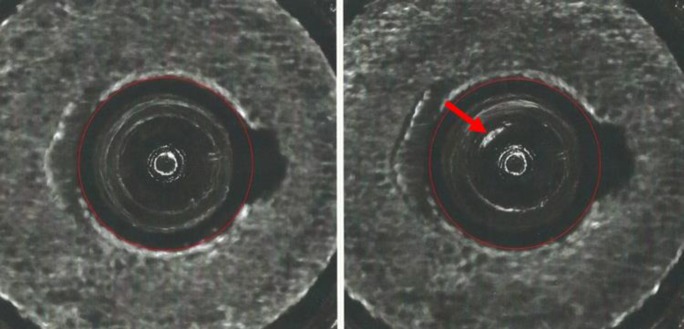
NIBIN image (right) corresponding to point d in Figs. 12 and 14 and a second image (left) obtained by the same operator on a different day that yielded a high correlation score when compared to the Golden Image for firing pin (Fig. 19, right).

**Fig. 21:**
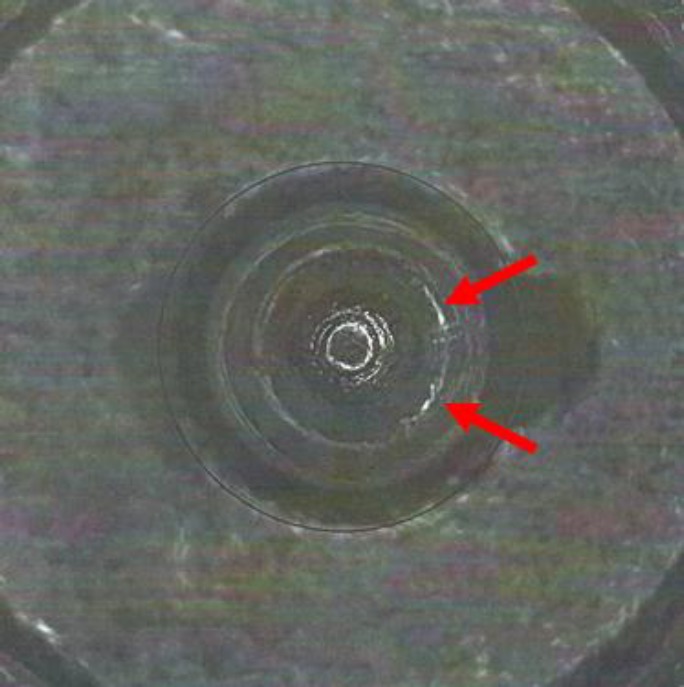
NIBIN image corresponding to point e in Figs. 12 and 14.

As shown in Figs. 12 and 14, there were four low correlation scores for images of firing pin impressions. Three of these (b, c, and d) were not included in the data to calculate a control limit. The fourth (e) seemed to be consistent with other firing pin data taken for the same station so it was included. Figures 18 and 21 show images for all four cases. The first three (b, c, and d) (Figs. 18 and 20) seem fairly consistent. All three firing pin images contain bright highlights, shown by the red arrows, whereas the Golden Image, shown on the right of Figs. 18 and 19, contains fairly continuous rings with more uniform illumination near the outer edge. The fourth image (e) yielded a slightly higher correlation score than the other three, and the extra highlights in the image of the firing pin impression are comparable to those of c and not as strong as those for b and d. Again, it is not clear whether an illumination, alignment, or focusing problem caused the spurious highlights in these images. Perhaps, a small amount of misalignment can enhance multiple reflections in the concave firing impression in such a way as to cause the bright highlights. Less likely, the highlights might arise from ambient illumination in the laboratory. Other images, taken before and after these did yield higher correlation scores, and samples of the images did not reveal the imaging anomalies shown here.

### 7.3 Ejector Marks

**Fig. 22:**
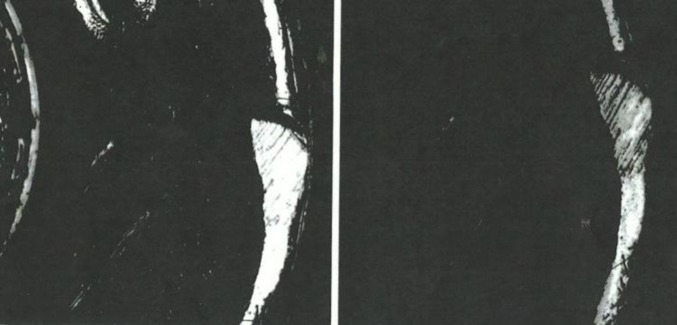
NIBIN image (left) corresponding to point f in Figs. 12 and 15 and Golden Image for ejector mark (right).

We also inspected the lowest scoring ejector mark image, which gave rise to point f in Figs. 12 and 15. The comparison with the Golden Image is shown in Fig. 22. It seems clear that there is misalignment between the acquired image and the Golden Image for ejector marks, a condition which likely led to the low correlation score.

## 8. Other Systematic Effects

### 8.1 Trading Places – Results from Two Stations

**Fig. 23:**
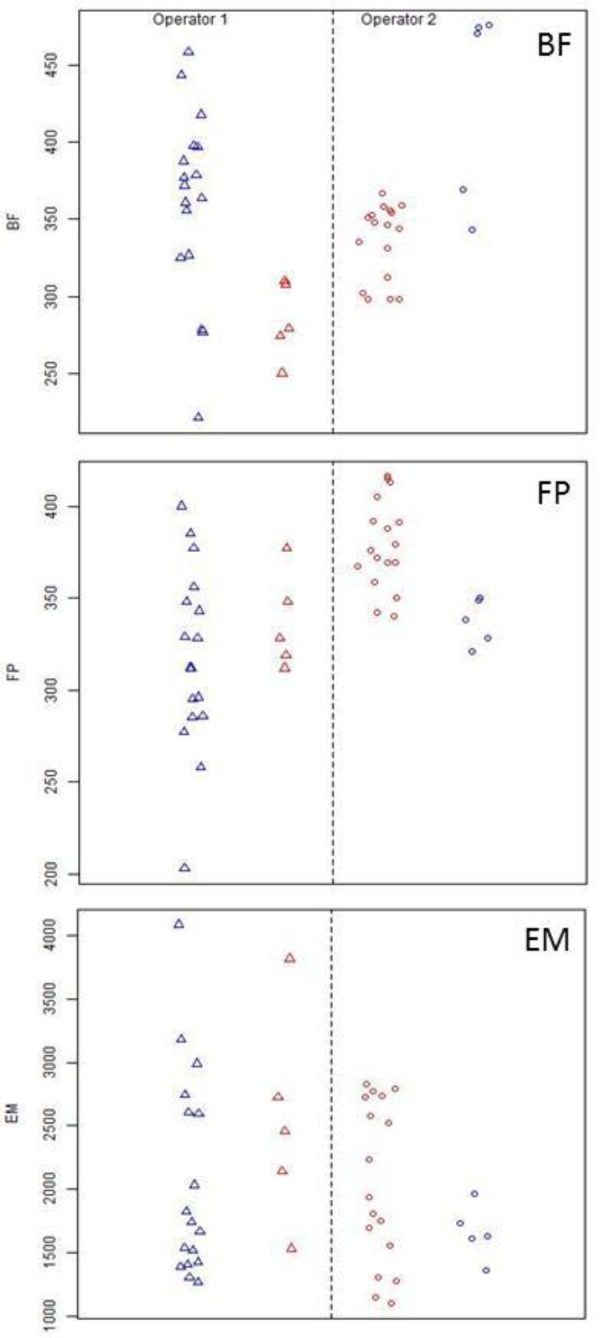
Operator and instrument effects for two instruments; blue – correlation results for instrument A, red – correlation results for instrument B. The data points are shifted laterally for clarity.

Figures 13 and 15 clearly indicate some significant differences in the results measured on different stations. It is not clear whether these differences arise from the operators, the instruments, the SRMs themselves, or a combination. For one laboratory, we were able to test for systematic effects arising in the instruments. Two of the operators used different instruments but the same unit of the SRM. Figure 23 shows all of the results (Phase 1 and Phase 2) for operator 1 using instrument A (blue triangles) and all of the results for operator 2 using instrument B (red circles). On average, the correlation results for firing pin are slightly higher for operator 2, and the variation in correlation scores for breech face and firing pin seem higher for operator 1. To assess whether differences in the instruments contributed to these differences, the operators traded places and performed five more acquisitions, which are represented by the red circles and the blue triangles. Overall, the differences are not very significant. Operator 2 may be slightly more consistent than operator 1 overall, and instrument B may be slightly more consistent than instrument A for breech face and firing pin correlations. This small sample of two instruments suggests that differences between the instruments can appear. However, these differences are folded into the proposed control limits because the results have been gathered from eleven different stations.

### 8.2 New versus Old Golden Images

**Fig. 24:**
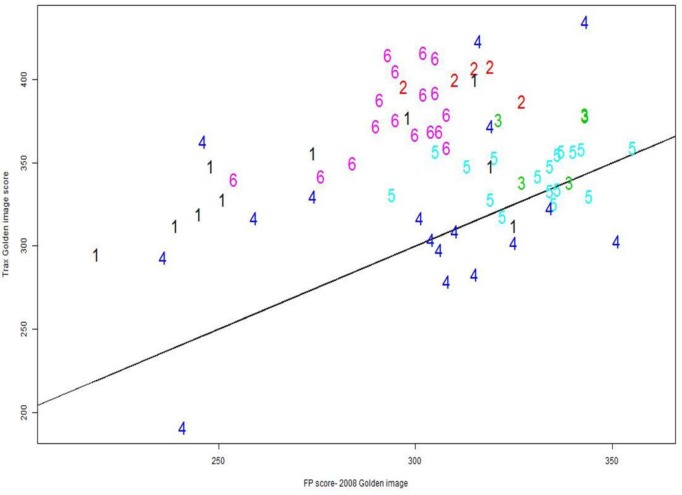
Comparison of firing pin correlation scores for two golden images; results for six participants, labelled 1–6.

The main question to explore for NBIC-2 was whether the control limits for BRASSTRAX stations should be changed from the control limits found for the IBIS Heritage stations during NBIC-1. We found (Table 1) that a relatively insignificant increase in the control limits for breech face and ejector mark is recommended for BRASSTRAX stations, but a significant increase in the control limit for firing pin is recommended, amounting to more than two standard deviations of the NBIC-2 data. We seek to learn the principal source of the difference between IBIS Heritage and BRASSTRAX technology, given that the correlation software version is the same for both and the images of both system versions are interoperable, that is, images for BRASSTRAX may be directly correlated with images for Heritage. Data for six NBIC-2 participants are shown in Fig. 24, which compares the firing pin correlation scores of the NBIC-2 images against the new BRASSTRAX Golden Image versus the correlation scores of the same NBIC-2 images against the original Heritage/NBIC-1 Golden Image. Most of the data lie above the 1:1 line, and the average correlation score for the NBIC-2 BRASSTRAX Golden Image is higher than that for the NBIC-1 (Heritage) Golden Image by approximately 46.

The principal physical difference between the two technologies is the pixel resolution. BRASSTRAX images contain (960 × 960) pixels, whereas Heritage images contain (480 × 480) pixels. Hence, the BRASSTRAX images may contain more significant detail than the Heritage images and the correlation of these details may result in higher correlation scores. This observation may be especially true for firing pin images which clearly occupy a smaller area on the camera frame than breech face images, as shown by Figs. 4 and 5. Hence, the lateral resolution of the Heritage images of firing pin impressions may be more limited by the camera pixel spacing than the BRASSTRAX images are. Carrying this argument further, we expect that the correlation of Heritage images with the BRASSTRAX Golden Image would score about the same as the correlation of BRASSTRAX images with the Heritage Golden Image and that the correlation of Heritage Images with the Heritage Golden Image should score even lower than those.

**Fig. 25:**
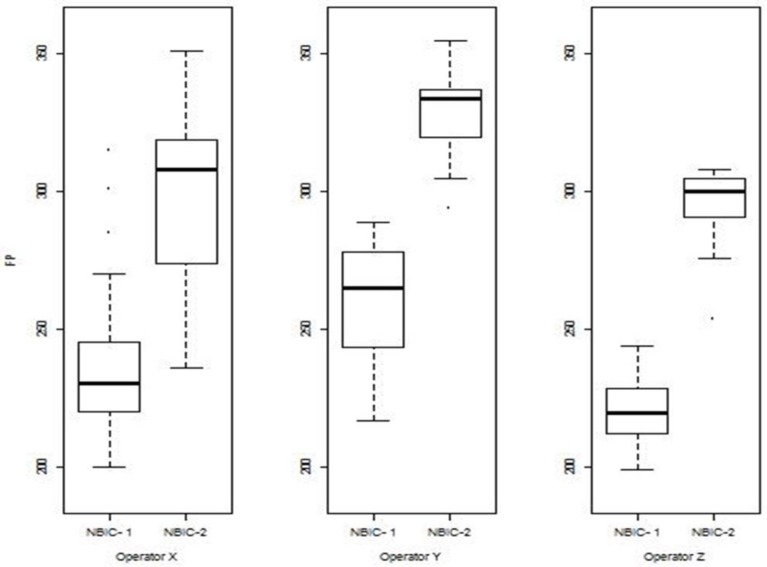
Comparison of firing pin correlation results for NBIC-1 and NBIC-2 acquisitions with respect to the Heritage Golden Image; results for three operators. The heavy solid horizontal lines inside the boxes represent the median values of the distributed data. The boxes represent the middle quartiles of the data. The bars outside the box show the high and low extent of the data, not including points considered to be outliers, which are shown separately.

The above observation about the importance of pixel resolution is supported by results where the NBIC-2 and NBIC-1 acquisitions taken by the three operators who participated in both projects are correlated with the NBIC-1 Golden Image. For firing pin impressions (Fig. 25), the NBIC-2 images produce significantly higher correlation scores than the NBIC-1 images. The NBIC-2 images have finer pixel spacing than the NBIC-1 images, and even though the Heritage Golden Image has a relatively coarse pixel spacing, the higher resolution NBIC-2 images may provide details that enhance the NIBIN correlation scores.

**Fig. 26:**
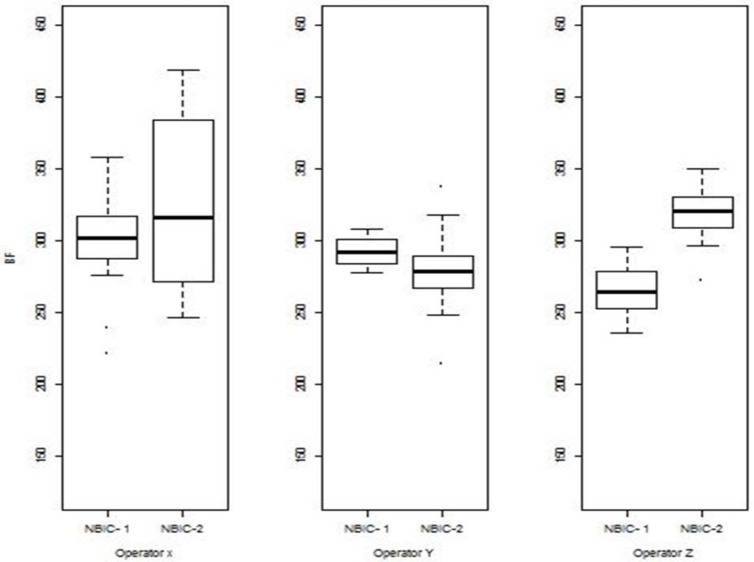
Comparison of breech face correlation results for NBIC-1 and NBIC-2 acquisitions with respect to the Heritage Golden Image; results for three operators.

**Fig. 27:**
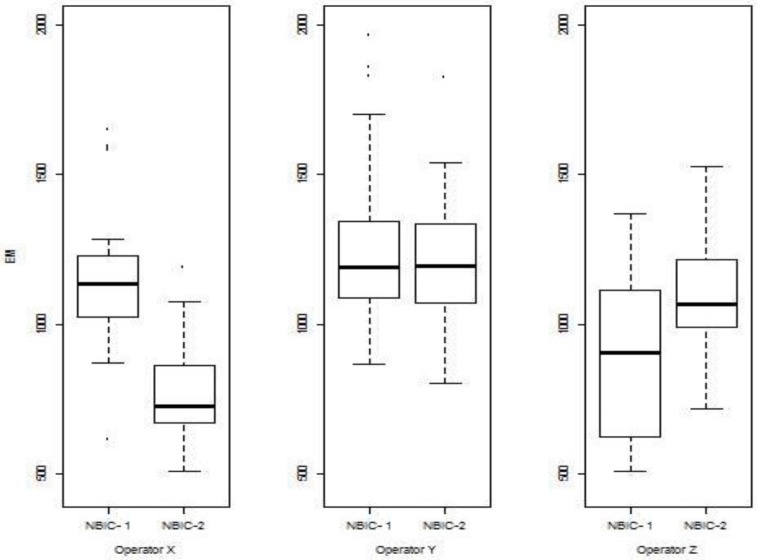
Comparison of Ejector Mark Correlation Results for NBIC-1 and NBIC-2 acquisitions with respect to the Heritage Golden Image; results for three operators.

This is not the case for breech face and ejector mark. Figures 26 and 27 do not show significantly higher correlation scores for either breech face or ejector mark with the NBIC-2 Golden Image than with the NBIC-1 Golden Image.

## 9. Measurement Procedure and Quality System for Cartridge Case Correlations

### 9.1 A Proposed Uncertainty Budget for Correlations of Breech Face and Firing Pin

Having discussed quality control issues for NIBIN stations, we come to the issue of the correlation scores themselves. On the one hand, the proprietary correlation software supplied to NIBIN by Ultra Electronics Forensic Technology is intended to yield only an ordered list of possible matches of images in the database to a subject cartridge case or bullet. IBIS BRASSTRAX scores are used for sorting possible candidate matches and are not meant to be interpreted as an estimate of an intrinsic physical quantity. On the other hand, it is natural to pose the question whether a large difference between two correlation scores likely indicates that one entry in the database matches significantly better to a subject cartridge case than another entry and a small difference indicates that two entries in the database have a similar quality of match to the subject cartridge case.

This question is essentially the same as determining the uncertainties of the IBIS scores. When is one IBIS score significantly different from another? We have a long way to go in this effort to evaluate all the sources of relative uncertainty for IBIS correlation scores, but we have developed procedures for evaluating two components of uncertainty. The Phase 2 data from NBIC-2 provides an estimate of the mean and variation of IBIS scores over a long period of time and from operator to operator for surfaces that are essentially identical. The average Phase 2 score for all NBIC-2 operators and stations for breech face was 329.1. The standard deviation was 52.5, or about 16.0 % of the average score. If we assume a strictly proportional model for uncertainty in BRASSTRAX correlation scores for breech face impressions, and if this Type A uncertainty is the dominant uncertainty component, then we can estimate the scores to have a relative standard uncertainty *f*_BF_ of 16.0 %. Furthermore, we can consider two scores to be significantly different at the one standard deviation level if their correlation scores for breech face differ by approximately 21 %. That is if the smaller correlation score is less than approximately 79 % of the larger score. A precise formula for this estimation is obtained from the equation:

$$S_{\mathrm{LA}}-S_{\mathrm{SM}}>{\left[{\left(f\;S_{\mathrm{LA}}\right)}^{2}+{\left(f\;S_{\mathrm{SM}}\right)}^{2}\right]}^{1/2},$$ (2)

where *S*_SM_ is the smaller correlation score, *S*_LA_ is the larger correlation score, and *f* is the relative standard uncertainty, 16.0 % in this case. This equation can be cast as a quadratic equation with *S*_SM_ as unknown. This leads to

$$S_{\mathrm{SM}}<S_{\mathrm{LA}}[1-{\left(2f^{2}-f^{4}\right)}^{1/2}]/(1-f^{2}).$$ (3)

For firing pin impressions, the average score from NBIC-2 was 344.3 and the standard deviation was 43.5, or 12.6 %. We might then consider two firing pin correlation scores to be significantly different if they differ by approximately 17 %, that is, if the smaller correlation score is less than approximately 83 % of the larger correlation score.

We do not expect nonlinear models to change these crude uncertainty estimates a great deal. Nevertheless, we tested for nonlinearity of variance using data for high-scoring non-matches. A few sets of results provide enough data for the calculation. These results were taken from firing pin impressions for a single high-scoring non-matching cartridge case acquired by six of the 14 participants. The calculated average correlation was 110.7 with a standard deviation of 9.5 (8.5 %). This result suggests that the relative standard deviation does not balloon into large values when the correlation signal decreases, and that non-linearity of the standard deviation is not significant.

**Fig. 28:**
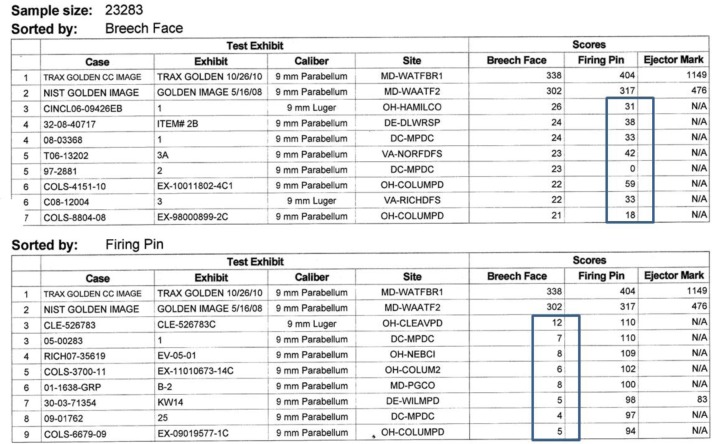
Example of non-matching scores for one acquisition. The highlighted scores are unordered non-matches.

What is more significant is the zero-level uncertainty, the correlation scores and their variations that result from correlating non-matching surfaces. This can be estimated from the NBIC-2 results as well by using the scores from a representative sample of non-matching surfaces, that is, from the scores that result when the SRM surfaces are matched with non-SRM surfaces in the database. We desire a random sample of these scores, so we do not use scores that are rank ordered. However, a random sample of non-matching scores is available from the NBIC-2 results. Figure 28 shows a table of results for one operator for one SRM entry. The upper table shows results ordered by breech face, with highest at the top. The firing pin scores, however, are not rank ordered. The correlation values for the entries that are obviously non-matching (31, 38, 33, 42, 0, 59, 33, 18) represent to us a small random sample of non-matching scores for firing pin. Likewise in the lower table, the scores (12, 7, 8, 6, 8, 5, 4, 5) represent a small random sample of non-matching scores for breech face.

When we average a large number of these correlation scores for the 12 operators of NBIC-2 for which we have such data, we arrive at a zero-level value for breech face of 7.78 ± 2.65 (1 standard deviation) and a zero-level value for firing pin of 31.4 ± 16.6 (1 standard deviation). These values imply for us that correlation scores close to these levels are not significantly different from a completely non-matching situation.

We take the criterion for a minimum meaningful correlation score to be a difference of one standard deviation from the zero-signal level. Accordingly, for both breech face and firing pin, we write the equation:

$$S_{\min}-S_{0}={\left[\sigma_{0}^{2}+{\left(f\;S_{\min}\right)}^{2}\right]}^{1/2},$$ (4)

where the term on the right hand side is the root-sum-square of two terms, the standard deviation of the zero signal and the estimated standard uncertainty of the minimum signal. Rounding up to the nearest integer value, the minimum meaningful correlation value for breech face (*S*minBF) is approximately 11 and the minimum meaningful correlation signal for firing pin (*S*minFP) is approximately 50. We summarize these results as

*f*_BF_ = 16.0%,
*f*_FP_ = 12.6%,
*S*_0BF_ = 7.8 ± 2.7,
*S*_0FP_ = 31 ± 17,
*S*_minBF_ = 11,
*S*_minFP_ = 50.

The above results apply for BRASSTRAX acquisitions. Similar results have been calculated for Heritage acquisitions and the values are:

*f*_BF_ = 13.6%,
*f*_FP_ = 13.3%,
*S*_0BF_ = 8.5 ± 2.8,
*S*_0FP_ = 27 ± 15,
*S*_minBF_ = 12,
*S*_minFP_ = 43.

For ejector marks, the relative variation of correlation scores is significantly larger than those for breech face and firing pin. The average score from NBIC-2 was 1470.3 and the standard deviation was 561.8, or 38.2 %. We might then consider two ejector mark correlation scores to be significantly different if they differ by approximately 44 %, that is, if the smaller correlation score is less than approximately 56 % of the larger correlation score. However, the distribution of ejector mark correlation scores is so widely distributed (Fig. 7), the model for uncertainty developed for breech face and firing pin is likely not appropriate for ejector marks.

### 9.2 Traceability Issues

The issue of measurement uncertainty within a quality system leads directly to the issue of measurement traceability. According to the International vocabulary of metrology – Basic and general concepts and associated terms (VIM) [21], metrological traceability is defined as:

“property of a measurement result whereby the result can be related to a reference through a documented unbroken chain of calibrations, each contributing to the measurement uncertainty.”

Although image acquisitions and correlations of cartridge cases in the NIBIN are not traceable to SI units, we now have a system of “para-traceability” for breech face impressions and firing pin impressions with

- the NIBIN Golden Images as standards,
- a comparison procedure generalized here as Appendix 1,
- a control chart with control limits that may be used to demonstrate consistent operation of individual NIBIN stations available on-line, see Appendix 2, and
- an uncertainty budget for correlations of breech face impressions and firing pin impressions, described in Sec. 9.1.

Overall, the quality system addresses the issues of

- demonstrating that individual NIBIN stations are operating properly and consistently with other stations in the system,
- detecting the presence of problems in operation,
- detecting significant differences in the correlation scores that may indicate which entries in the NIBIN database have significantly higher potential than others for matching a subject piece of evidence.

## 10. Conclusion

The main question to explore for NBIC-2 was whether the control limits for BRASSTRAX systems should be changed from the control limits found for the IBIS Heritage systems during NBIC-1. The NBIC-2 results, shown in Sec. 6, yield control limits that are not significantly higher than the NBIC-1 control limits for breech face and ejector mark acquisitions but yield a control limit for firing pin acquisitions that is about two standard deviations higher.

The quality procedure discussed in Sec. 9 comprises a baseline of best practices, formalized by control limits, with control charts for monitoring individual performance. It was developed and tested for the acquisition stations in ATF’s NIBIN, but it can be adapted for other types of automated inspection stations for cartridge cases. Whatever the operating system, operators can use the SRMs together with control charts to monitor the performance consistency of themselves and their stations. The NBIC-2 project dealt only with cartridge cases because BRASSTRAX stations in NIBIN were designed solely for the measurement of cartridge cases and are not capable of measuring bullets.

Current automated ballistics identification systems are primarily based on image comparisons using optical microscopy. The correlation accuracy depends on image quality, which is largely affected by lighting conditions. This effect was highlighted in the NBIC-1 project [10]. Because ballistic signatures are geometrical micro-topographies by nature, direct measurement and correlation of the surface topography itself has been proposed for ballistics identification [22,23], and has achieved favorable initial results for bullet identifications from profile signatures [24]. Several commercial systems for topography measurement and correlation of bullets and cartridge cases have also been developed [for examples see 3,25,26, 27]. We are currently working on the development of a 3D ballistics identification system using topography measurements and a new correlation method based on the integration of correlations from small surface areas [28].

NIST SRM 2460/2461 Standard Bullets and Cartridge Cases function as reference standards for establishing traceability for both topography measurements at NIST and image correlations of NIBIN. For the topography measurements, the measurement traceability is established using the topography images of SRM cartridge cases and bullets and NIST-measured parameters, *D*_s_ and *CCF*_max_, [14,15]. For the image acquisitions of NIBIN, traceability of acquisition of breech face impressions and firing pin impressions is supported by correlation of SRM bullet and cartridge case images at local IBIS sites with respect to the Golden Images of the National Laboratory Center of ATF.

Because the NIBIN correlations for ejector mark images and bullet images are more variable, we have not yet developed an uncertainty budget for those regions of interest, although we have proposed control limits for correlation of the acquisitions of the SRM 2461 ejector marks and the SRM 2460 bullets. The SRM Cartridge Cases and Bullets, when used with the control charts and control limits, are powerful tools for quality assurance of NIBIN acquisitions and correlations.

## Figures and Tables

**Fig. 10:**
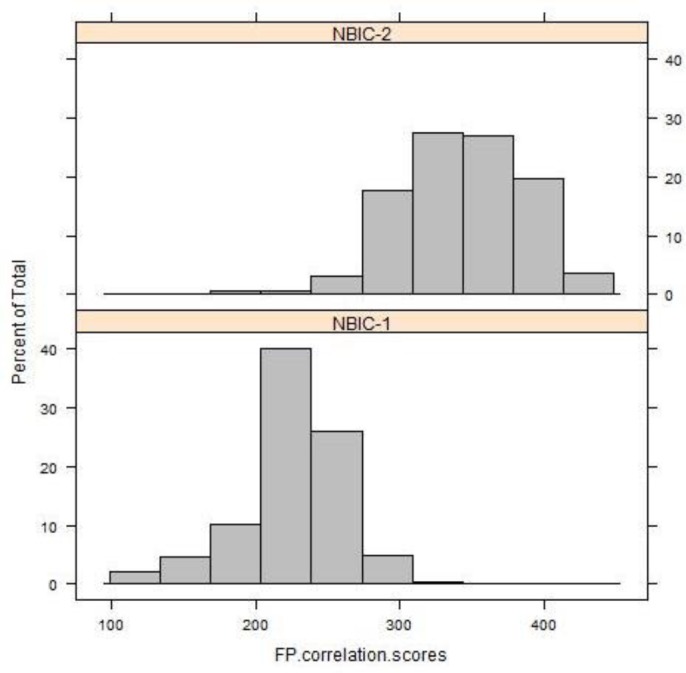
Histograms of correlation scores for SRM 2461 firing pin impressions for NBIC-1 and NBIC-2.

**Fig. 11:**
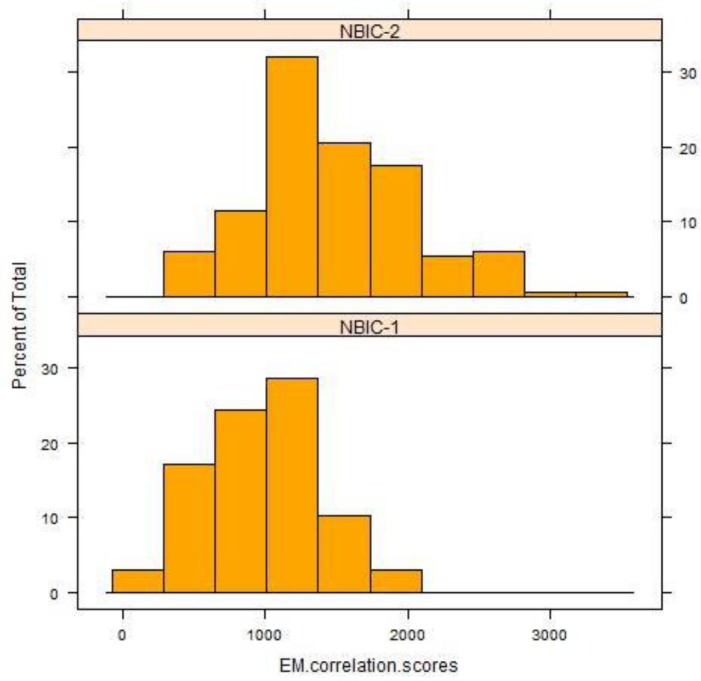
Histograms of correlation scores for SRM 2461 ejector marks for NBIC-1 and NBIC-2.

**Table 1:** Control limits for breech face, firing pin and ejector mark BRASSTRAX correlation scores of SRM cartridge cases with a one sided 95 % confidence level. The values are unitless.

	Mean	Standard deviation	95 % Lower control limit	Control limit from NBIC-1
Breech face	329	53	243	221
Firing pin	344	44	273	171
Ejector mark	1476	562	552	400

## Footnotes

1 Certain commercial equipment, instruments, or materials are identified in this paper to specify adequately the experimental procedure. Such identification does not imply recommendation or endorsement by the National Institute of Standards and Technology, nor does it imply that the materials or equipment identified are necessarily the best available for the purpose.

## 11. Appendix 1: Developing a Control Chart for Automated Ballistics Acquisition Systems Using NIST SRM 2461 Standard Cartridge Cases

The NIST SRM Standard Cartridge Case is intended for quality testing of automated acquisitions of cartridge case breech face impressions, firing pin impressions, and ejector marks. The first control charts were developed for bullet and cartridge case acquisitions for the ATF’s NIBIN, which uses the IBIS Heritage and IBIS BRASSTRAX-3D acquisition stations and system correlation software. The standards are Golden Images developed during the NBIC and NBIC-2 projects, which reside on a NIBIN server housed at the ATF National Laboratory Center. Control limits were proposed for both types of acquisition technology after two thorough periods of testing and correlations by a number of IBIS operators.

While the control charts were created based on NBIC data, the statistical methods are straightforward and can be adapted for any type of automated inspection station. The main change would be the value of the control limit, which depends on the software used to calculate correlation. A brief description follows for developing a control chart on systems that do not have access to the NIBIN Golden Images. The proposed procedure contains general steps for acquisition of breech face impressions, firing pin impressions, and ejector marks on cartridge cases using SRM 2461. A sample control chart (see Appendix 2) is also included. Control charts for bullet acquisitions using SRM 2460 could be developed in a similar way.

1. Follow standard procedures from the manufacturer for maintenance and setup of the inspection station and for calibration check at turn on.
2. Enter case and exhibit ID information according to procedures described by the manufacturer of the station. Noteworthy information requirements are the date of the image acquisition and the identity of the SRM, for example, “SRM 2461-XXX,” where “XXX” represents the serial number.
3. Follow the manufacturer’s procedure for acquiring a cartridge case. For most conditions, use the same procedure as that for automated imaging of fired cartridge cases.
4. Create “master image” acquisitions for the breech face, firing pin, and ejector mark by some suitable approach, such as using ideal illumination conditions or loading an ideal image developed elsewhere by the manufacturer of the station or by another user of a comparable station. Subsequent images for breech face, firing pin, and ejector mark will be correlated with respect to the master images.
5. Calculate an estimate for a fixed lower control limit (fixed CL) for the correlation scores for each region, based on previous observations or on the experiences of other experts operating similar systems. The fixed CL should be chosen so that approximately 95 % of the correlation readings are expected to lie above it. If a correlation result falls below the fixed CL, the operator should investigate if a measurement problem is developing.
6. Perform image acquisitions of the breech face impression, firing pin impression, and ejector mark on the SRM 2461.
7. Correlate each type of acquired image (breech face, firing pin, ejector mark) with the appropriate master image and calculate the correlation score according to the manufacturer’s instructions. Enter these scores on the control chart.
8. Continue to perform acquisitions of SRM 2461 using the procedure above at appropriate intervals imposed by your quality system. It is recommended that an acquisition of a standard cartridge case be entered at least once per month to verify the proper operation of the station. However, you should refer to your own laboratory’s policy and procedure guidelines for the frequency of acquisition. The cartridge case acquisitions should also be entered into the system during each software and hardware upgrade as well as after any scheduled or unscheduled maintenance. All entries and results should be documented.
9. After at least two sets of correlations have been taken, calculate a mean (*µ*), a standard deviation (*σ*), and a dynamic lower control limit (dynamic CL). The dynamic CL may be calculated from
  dynamicCL=μ−1.645σ.
  This quantity represents a 95 % one sided lower confidence interval for a Gaussian distribution. The mean and the dynamic CL should fluctuate slightly with each successive data point and should settle into a stable value after about ten readings.
10. The correlation scores for the breech face impression, firing pin impression, and ejector mark should all be higher than both the fixed CL described in Step 5 and the dynamic CL calculated in Step 8. If not, after an appropriate number of records (for example 10 records) determine a new fixed CL value based on the updated statistical information and on a consensus of the lab personnel. The fixed CL value may change in the future as the software or hardware changes.
11. Once the laboratory has established a stable value for its fixed control limit, maintain plots of the correlation scores on control charts to demonstrate that the acquisitions are being performed consistently and with high correlation to the master image. NIST’s spreadsheet (Appendix 2) automatically provides a plot (Fig. 6) of each measured correlation value and the fixed CL versus the date of the measurement, as well as a running plot of the dynamic CL [17] for each cartridge case region of interest. This is done as long as at least two entries are made on the top sheet.
12. If any of the correlation scores drops below the fixed CL value, flag the results and the date, then double check the alignment and reacquire the cartridge case images.
13. If the correlation score still remains below the fixed CL value, you may want to have another trained IBIS user acquire the cartridge case using the station. If the second score is above the fixed CL value, then you may have a user issue. If the score is still low, then you may be observing a change in the station performance that needs to be diagnosed. Check the image acquisition procedure. This may include checking the basic set up and calibration of the acquisition station, as well as the alignment of the cartridge case.
14. Handling and Cleaning Procedure: The standard cartridge cases are expected to be robust and to maintain their quality over many years. However, it is good procedure to avoid handling the surface of the head stamp area in order to avoid unnecessary scratches and finger contamination from marring it. Likewise, cleaning should also be avoided as much as possible because the cleaning process itself can introduce irreversible changes in the surface topography of the cartridge case. If it is clear that contamination has been introduced on the surface to the extent that it has been visibly changed, then a mild cleaning procedure may be used. The suggested procedure is to clean only the contaminated area with a lab swab/cotton tip applicator moistened with ethyl alcohol.
