# Supplementary material for: The Second National Ballistics Imaging Comparison (NBIC-2)
Source: J Res Natl Inst Stand Technol. 2015 Jan 8;119:644–73. doi: 10.6028/jres.119.028 (PMC4487285; doi:10.6028/jres.119.028)
Supplement: Supplementary file 1 [file jres.119.028c.pdf]

Volume 119 (2014) http://dx.doi.org/10.6028/jres.119.028.c
Journal of Research of the National Institute of Standards and Technology

# *Control Chart for Ballistic Acquisition and Correlation Systems*

**T. V. Vorburger, J. Yen, J. F. Song, R. M. Thompson, T. B. Renegar, A. Zheng, M. Tong**

National Institute of Standards and Technology,
Gaithersburg, MD 20899

**and**

**M. Ols**

Bureau of Alcohol, Tobacco, Firearms and Explosives (ATF),
Ammendale, MD 20705

theodore.vorburger@nist.gov
james.yen@nist.gov
jun-feng.song@nist.gov
robert.m.thompson@nist.gov
thomas.renegar@nist.gov
alan.zheng@nist.gov
mingsi.tong@nist.gov
martin.ols@atf.gov

The supplemental Control Chart linked in Sec. 12 of "The Second National Ballistics Imaging Comparison (NBIC-2)" is embedded in this pdf. To access the .xlsm file please follow these directions.

1) Save the file to disc and open it using Adobe Acrobat or Adobe Reader.[1]
2) Open the "Attachments" pane by clicking on the button that looks like a paper clip.
3) Right-click on the file name and select "Open Attachment.
4) If prompted by the PDF reader, select "Open this file" and "OK" to open the file.

[1] Certain commercial products are identified in this file in order to specify the procedures adequately. Such identification is not intended to imply recommendation or endorsement by the National Institute of Standards and Technology, nor is it intended to imply that the products identified are necessarily the best available for the purpose.
